# Supplementary material for: Injury Occurrence and Return to Dance in Professional Ballet: Prospective Analysis of Specific Correlates
Source: Int J Environ Res Public Health. 2019 Mar 3;16(5):765. doi: 10.3390/ijerph16050765 (PMC6427676; doi:10.3390/ijerph16050765)
Supplement: Supplementary file 1 [file ijerph-16-00765-s001.zip › ijerph-452640-suppl xml.pdf]

Table S1. Descriptive data for the observed variables (F–frequencies, %–percentage).

	All (n = 99)		Males (n = 41)		Females (n = 58)	
	F	%	F	%	F	%
**Age**						
<19 years	9	9.09	5	12.20	4	6.90
19–22 years	14	14.14	6	14.63	8	13.79
23–26 years	12	12.12	6	14.63	6	10.34
27–30 years	10	10.10	3	7.32	7	12.07
31–34 years	10	10.10	3	7.32	7	12.07
35–38 years	15	15.15	9	21.95	6	10.34
>38 years	29	29.29	9	21.95	20	34.48
**Educational level**						
Elementary school	4	4.04	2	4.88	2	3.45
High school	59	59.60	25	60.98	0	0.00
College/University student	1	1.01	1	2.44	34	58.62
College/University level	35	35.35	13	31.71	22	37.93
**Experience in ballet**						
10–15 years	22	22.22	13	31.71	9	15.52
16–20 years	21	21.21	6	14.63	15	25.86
21–25 years	26	26.26	13	31.71	13	22.41
>25 years	30	30.30	9	21.95	21	36.21
**Ballet performance level**						
Corps de ballet	19	19.19	5	12.20	14	24.14
First artist	77	77.78	34	82.93	43	74.14
Soloist	3	2.00	1	2.50	1	1.72
Principal	1	1.00	1	2.50	0	0.00
**Number of training hours**						
<20 hours	10	10.10	4	9.76	6	10.34
21–25 hours	21	21.21	5	12.20	12	20.69
26–30 hours	23	23.23	10	24.39	13	22.41
>30 hours	45	45.45	22	53.66	27	46.55
**Cigarette smoking**						
Never smoked	44	44.44	15	36.59	29	50.00
Quit	1	1.01	0	0.00	1	1.72
From time to time, but not daily	10	10.10	0	0.00	10	17.24
Less than 10 cigs daily	10	10.10	2	4.88	8	13.79
10–20 cigs daily	26	26.26	18	43.90	8	13.79
From one to two packs daily	8	8.08	6	14.63	2	3.45
More than 2 packs daily	0	0.00	0	0.00	0	0.00
**Binge drinking**						
I don't drink alcohol	9	9.09	8	19.51	1	1.72
I drink alcohol but never binge	26	26.26	8	19.51	18	31.03
Rarely	12	12.12	5	12.20	7	12.07
Binge drinking couple of times per year	6	6.06	2	4.88	4	6.90
Binge drinking once a month or so	29	29.29	9	21.95	20	34.48
Binging one a week	8	8.08	3	7.32	5	8.62
Binging couple of times per week	9	9.09	6	14.63	3	5.17
**Consumption of illicit drugs**						
Yes	26	26.26	10	24.39	16	27.58
No	73	73.73	31	75.61	42	72.41
**Number of injuries**						
0	23	23.23	9	21.95	14	24.14
1	29	29.29	12	29.27	17	29.31
2	22	22.22	6	14.63	16	27.59
3	6	6.06	2	4.88	4	6.90
4	9	9.09	5	12.20	4	6.90
4	3	3.03	2	4.88	1	1.72
5	4	4.04	2	4.88	2	3.45
6	0	0.00	0	0.00	0	0.00
7	0	0.00	0	0.00	0	0.00
8	0	0.00	0	0.00	0	0.00
9	3	3.03	3	7.32	0	0.00
**Time-off from injury**						

No absence	37	37.37	13	31.71	24	41.38
Less than 3 days	14	14.14	8	19.51	6	10.34
4–7 days	3	3.03	2	4.88	1	1.72
More than 7 days	45	45.45	18	43.90	27	46.55
