# Supplementary material for: Injury Occurrence and Return to Dance in Professional Ballet: Prospective Analysis of Specific Correlates
Source: Int J Environ Res Public Health. 2019 Mar 3;16(5):765. doi: 10.3390/ijerph16050765 (PMC6427676; doi:10.3390/ijerph16050765)
Supplement: Supplementary file 1 [file ijerph-16-00765-s001.zip › ijerph-452640-suppl xml.docx]

**Table S1.** Descriptive data for the observed variables (F–frequencies, %–percentage).

|  | **All (*n* = 99)** | | **Males (*n* = 41)** | | **Females (*n* = 58)** | |
| --- | --- | --- | --- | --- | --- | --- |
|  | **F** | **%** | **F** | **%** | **F** | **%** |
| **Age** |  |  |  |  |  |  |
| <19 years | 9 | 9.09 | 5 | 12.20 | 4 | 6.90 |
| 19–22 years | 14 | 14.14 | 6 | 14.63 | 8 | 13.79 |
| 23–26 years | 12 | 12.12 | 6 | 14.63 | 6 | 10.34 |
| 27–30 years | 10 | 10.10 | 3 | 7.32 | 7 | 12.07 |
| 31–34 years | 10 | 10.10 | 3 | 7.32 | 7 | 12.07 |
| 35–38 years | 15 | 15.15 | 9 | 21.95 | 6 | 10.34 |
| >38 years | 29 | 29.29 | 9 | 21.95 | 20 | 34.48 |
| **Educational level** |  |  |  |  |  |  |
| Elementary school | 4 | 4.04 | 2 | 4.88 | 2 | 3.45 |
| High school | 59 | 59.60 | 25 | 60.98 | 0 | 0.00 |
| College/University student | 1 | 1.01 | 1 | 2.44 | 34 | 58.62 |
| College/University level | 35 | 35.35 | 13 | 31.71 | 22 | 37.93 |
| **Experience in ballet** |  |  |  |  |  |  |
| 10–15 years | 22 | 22.22 | 13 | 31.71 | 9 | 15.52 |
| 16–20 years | 21 | 21.21 | 6 | 14.63 | 15 | 25.86 |
| 21–25 years | 26 | 26.26 | 13 | 31.71 | 13 | 22.41 |
| >25 years | 30 | 30.30 | 9 | 21.95 | 21 | 36.21 |
| **Ballet performance level** |  |  |  |  |  |  |
| Corps de ballet | 19 | 19.19 | 5 | 12.20 | 14 | 24.14 |
| First artist | 77 | 77.78 | 34 | 82.93 | 43 | 74.14 |
| Soloist | 3 | 2.00 | 1 | 2.50 | 1 | 1.72 |
| Principal | 1 | 1.00 | 1 | 2.50 | 0 | 0.00 |
| **Number of training hours** |  |  |  |  |  |  |
| <20 hours | 10 | 10.10 | 4 | 9.76 | 6 | 10.34 |
| 21–25 hours | 21 | 21.21 | 5 | 12.20 | 12 | 20.69 |
| 26–30 hours | 23 | 23.23 | 10 | 24.39 | 13 | 22.41 |
| >30 hours | 45 | 45.45 | 22 | 53.66 | 27 | 46.55 |
| **Cigarette smoking** |  |  |  |  |  |  |
| Never smoked | 44 | 44.44 | 15 | 36.59 | 29 | 50.00 |
| Quit | 1 | 1.01 | 0 | 0.00 | 1 | 1.72 |
| From time to time, but not daily | 10 | 10.10 | 0 | 0.00 | 10 | 17.24 |
| Less than 10 cigs daily | 10 | 10.10 | 2 | 4.88 | 8 | 13.79 |
| 10–20 cigs daily | 26 | 26.26 | 18 | 43.90 | 8 | 13.79 |
| From one to two packs daily | 8 | 8.08 | 6 | 14.63 | 2 | 3.45 |
| More than 2 packs daily | 0 | 0.00 | 0 | 0.00 | 0 | 0.00 |
| **Binge drinking** |  |  |  |  |  |  |
| I don't drink alcohol | 9 | 9.09 | 8 | 19.51 | 1 | 1.72 |
| I drink alcohol but never binge | 26 | 26.26 | 8 | 19.51 | 18 | 31.03 |
| Rarely | 12 | 12.12 | 5 | 12.20 | 7 | 12.07 |
| Binge drinking couple of times per year | 6 | 6.06 | 2 | 4.88 | 4 | 6.90 |
| Binge drinking once a month or so | 29 | 29.29 | 9 | 21.95 | 20 | 34.48 |
| Binging one a week | 8 | 8.08 | 3 | 7.32 | 5 | 8.62 |
| Binging couple of times per week | 9 | 9.09 | 6 | 14.63 | 3 | 5.17 |
| **Consumption of illicit drugs** |  |  |  |  |  |  |
| Yes | 26 | 26.26 | 10 | 24.39 | 16 | 27.58 |
| No | 73 | 73.73 | 31 | 75.61 | 42 | 72.41 |
| **Number of injuries** |  |  |  |  |  |  |
| 0 | 23 | 23.23 | 9 | 21.95 | 14 | 24.14 |
| 1 | 29 | 29.29 | 12 | 29.27 | 17 | 29.31 |
| 2 | 22 | 22.22 | 6 | 14.63 | 16 | 27.59 |
| 3 | 6 | 6.06 | 2 | 4.88 | 4 | 6.90 |
| 4 | 9 | 9.09 | 5 | 12.20 | 4 | 6.90 |
| 4 | 3 | 3.03 | 2 | 4.88 | 1 | 1.72 |
| 5 | 4 | 4.04 | 2 | 4.88 | 2 | 3.45 |
| 6 | 0 | 0.00 | 0 | 0.00 | 0 | 0.00 |
| 7 | 0 | 0.00 | 0 | 0.00 | 0 | 0.00 |
| 8 | 0 | 0.00 | 0 | 0.00 | 0 | 0.00 |
| 9 | 3 | 3.03 | 3 | 7.32 | 0 | 0.00 |
| **Time-off from injury** |  |  |  |  |  |  |
| No absence | 37 | 37.37 | 13 | 31.71 | 24 | 41.38 |
| Less than 3 days | 14 | 14.14 | 8 | 19.51 | 6 | 10.34 |
| 4–7 days | 3 | 3.03 | 2 | 4.88 | 1 | 1.72 |
| More than 7 days | 45 | 45.45 | 18 | 43.90 | 27 | 46.55 |
